# Axillary Web Syndrome in Breast Cancer Women: What Is the Optimal Rehabilitation Strategy after Surgery? A Systematic Review

**DOI:** 10.3390/jcm11133839

**Published:** 2022-07-01

**Authors:** Lorenzo Lippi, Alessandro de Sire, Luigi Losco, Kamal Mezian, Arianna Folli, Mariia Ivanova, Lorenzo Zattoni, Stefano Moalli, Antonio Ammendolia, Carmine Alfano, Nicola Fusco, Marco Invernizzi

**Affiliations:** 1 Physical and Rehabilitative Medicine, Department of Health Sciences, University of Eastern Piedmont “A. Avogadro”, 28100 Novara, Italy; lorenzolippi.mt@gmail.com (L.L.); arianna.folli23@gmail.com (A.F.); stefano.moalli@libero.it (S.M.); marco.invernizzi@med.uniupo.it (M.I.); 2 Dipartimento Attività Integrate Ricerca e Innovazione (DAIRI), Translational Medicine, Azienda Ospedaliera SS. Antonio e Biagio e Cesare Arrigo, 15121 Alessandria, Italy; 3 Physical and Rehabilitative Medicine Unit, Department of Medical and Surgical Sciences, University of Catanzaro “Magna Graecia”, Viale Europa, 88100 Catanzaro, Italy; ammendolia@unicz.it; 4 Plastic Surgery Unit, Department of Medicine, Surgery and Dentistry, University of Salerno, Via Salvador Allende, 43, Baronissi, 84081 Salerno, Italy; luigi.losco@gmail.com (L.L.); calfano@unisa.it (C.A.); 5 Department of Rehabilitation Medicine, First Faculty of Medicine, Charles University and General University Hospital, 11000 Prague, Czech Republic; kamal.mezian@gmail.com; 6 Division of Pathology, IEO, European Institute of Oncology IRCCS, Via Giuseppe Ripamonti 435, 20141 Milan, Italy; mariia.ivanova@ieo.it (M.I.); lorenzo.zattoni@unimi.it (L.Z.); nicola.fusco@unimi.it (N.F.); 7 Department of Oncology and Hemato-Oncology, University of Milan, Via Festa del Perdono 7, 20122 Milan, Italy

**Keywords:** axillary web syndrome, breast cancer, rehabilitation, pain management, quality of life

## Abstract

Background: Axillary web syndrome (AWS) is one of the most prevalent and underrecognized disorders affecting breast cancer (BC) women. However, the optimal therapeutic strategy to manage AWS is far from being fully characterized. Therefore, this systematic review aims to provide a broad overview of the available rehabilitation treatments in this burdensome condition. Methods: On 13 January 2022, PubMed, Scopus, Web of Science, Cochrane, and PEDro were systematically searched for clinical studies assessing rehabilitation interventions in post-surgical BC women with AWS. The outcomes analyzed were pain, AWS clinical resolution, upper limb function, and health-related quality of life (HR-QoL). Results: The search identified 1115 records, of which 11 studies were included. A total of 174 patients were assessed (ages ranging from 37 and 66 years old). The interventions included manual lymphatic drainage, manual therapy, stretching, resistance training, mobilization techniques, and Kinesio tape. Positive improvements were reported in terms of pain relief (in 7 studies), AWS clinical resolution (in 9 studies), upper limb function (in 10 studies), and HR-QoL (in 2 studies). Conclusions: Our findings suggest that rehabilitation might be considered an effective therapeutic strategy in AWS patients. Further RCTs are needed to characterize the optimal rehabilitative interventions.

## 1. Introduction

Breast cancer (BC) is the most common female tumor and one of the most common causes of death worldwide [1,2]. However, the growing efforts in early diagnosis and the recent advances in cancer treatments have consistently improved the overall survival of the disease [3]. Although survival represents the primary outcome of cancer treatments, in recent years, growing attention has been paid to physical and psychosocial sequelae of BC treatment affecting the wellbeing and health-related quality of life (HR-QoL) of long-term cancer survivors [4]. BC survivors might experience several underestimated and understudied complications, including post-traumatic stress disorders [5], breast cancer-related lymphedema (BCRL) [6], BC fatigue [7,8], aromatase inhibitor-induced musculoskeletal syndrome [9], cancer treatment-induced bone loss [10], chemo-induced peripheral neuropathy [11], and axillary web syndrome (AWS) [12,13].

AWS is one of the least studied negative sequelae affecting a substantial number of BC survivors after surgical procedures [14]. AWS was first defined by Moskovitz et al. [12] in 2001 as a painful cording characterized by a palpable and/or visible web of string-like structures typically localized subcutaneously at the axilla homolateral to the breast surgery [12]. Other common AWS sites include the arm, forearm, and wrist [15,16,17,18]. Its prevalence varies deeply, ranging from 6% to 91% [19], although it is usually considered fairly common [20], and a growing number of papers have been focused on this disabling sequela affecting BC HR-QoL in the last twenty years. 

Besides pain, BC patients suffering from AWS might be affected by a significant functional limitation due to range-of-motion limitations of the shoulder, in particular in flexion and abduction, with detrimental effects on HR-QoL [15,21,22]. Several risk factors involved in AWS onset have been identified, including axillary lymph node dissection (ALND), the number of lymph nodes removed, and the extent of axillary surgery [20,23,24]. On the other hand, several questions on the pathological mechanisms underpinning AWS onset are still unsolved [14]. In particular, it has been suggested that axillary surgery might induce thrombosis in lymphatic vessels [14]. In contrast, Moskovitz et al. suggested that both lymphatic vessels and blood vessels might be involved in AWS etiology [12]. 

Concurrently, recent research proposed that tumor-specific biological features might have a significant role in the onset of several BC-related complications, including AWS, and might be crucial to implementing a precise risk stratification and tailored preventive strategies [25]. However, at present, strong evidence supporting the preventive management of AWS is still lacking, and there is no consensus about the optimal therapeutic approach to this troublesome condition [26]. 

In this scenario, rehabilitation might positively impact AWS prevention, pain management, and AWS treatment after BC surgery [27]. However, despite the wide variability of rehabilitative approaches proposed in the current literature, to date, the optimal rehabilitation strategies to manage AWS are far from being fully understood [26].

Therefore, this systematic review aims to map the current literature and provide an overview of the rehabilitation treatments for AWS to provide relevant clinical hints and guide future research in the identification of the optimal management of this disabling disease. 

## 2. Materials and Methods

### 2.1. Search Strategy

This systematic review was performed following the Preferred Reporting Items for Systematic Reviews and Meta-Analyses (PRISMA) statement (PROSPERO registration number: CRD42022328641) [28]. 

On 13 January 2022, two investigators independently searched the databases. We selected and systematically searched PubMed/Medline, Scopus, Web of Science, Cochrane Central Register of Controlled Trials (CENTRAL), and Physiotherapy Evidence Database (PEDro) for manuscripts published up to the search date. The search strategy is described in detail in Table 1.

### 2.2. Selection Criteria 

In accordance with the PICO model [29], we considered eligible studies satisfying the following criteria:

- (P) Participants: Adult women suffering from AWS after breast cancer surgery. Studies assessing patients with a diagnosis of AWS by clinical examination or ultrasound assessment were included (without restrictions in terms of AWS diagnosis).
- Intervention: Rehabilitation treatment (education, physiotherapy, therapeutic exercise, myofascial relaxation techniques, scar treatment, manual lymphatic drainage (MLD), and physical therapies). We did not include studies involving pharmacological therapy in AWS management unless it was combined with rehabilitation treatment.
- (C) Comparator: any comparator, including placebo, pharmacological treatment, non-pharmacological treatment, or no treatment.
- (O) Outcome: The primary outcome was self-reported pain. The secondary outcomes were AWS resolution of the clinical presentation, upper limb function, and HR-QoL.

We included manuscripts published in peer-reviewed international journals in the English language. No restrictions on study design were applied. The exclusion criteria were: (i) language other than English; (ii) conference abstracts; (iii) studies involving animals; (iv) systematic reviews and meta-analyses. 

After duplicate removal, two investigators (AdS, LLi) independently reviewed the title and abstracts of the retrieved articles to identify relevant articles. Any discordance was resolved by collegial discussion. If consensus was not achieved, a third reviewer (MIn) was consulted. As an additional source, the reference lists of the included studies were searched for relevant records. 

Lastly, relevant records were then assessed in full text by two reviewers (AdS, LLi); any cases of disagreement were resolved by consulting a third reviewer (MIn).

### 2.3. Data Extraction and Synthesis

All data were assessed and extracted by two authors (AdS, LLi) independently from full-text documents into Excel. Any disagreement between the two reviewers was resolved by collegial discussion among the authors. In case of disagreement, a third author (MIn) was consulted. 

The following data were extracted: (1) title; (2) authors; (3) publication year; (4) country of origin; (5) study design; (6) participant characteristics (number, mean age, body mass index (BMI)); (7) breast surgery characteristics (time-from-surgery, type of surgery); (8) interventions’ characteristics; (9) comparator characteristics (when applicable); (10) outcomes (both primary and secondary); (11) main findings; (12) follow-up (when applicable). 

The data extracted were summarized in tables through a qualitative synthesis.

### 2.4. Quality Assessment 

The study quality was assessed through the checklist of the Joanna Briggs Institute Critical Appraisal Checklist for Quasi-Experimental Studies (JBI-QES) (non-randomized experimental studies) [30]. Each article was assessed independently by two authors (MIn), and any disagreement was resolved by a third author (MIn). The JBI-QES tool includes nine different domains assessing the risk of bias: Question Q1 = Is it clear in the study what is the ‘cause’ and what is the ‘effect’?; Q2 = Were the participants included in any comparisons similar?; Q3 = Were the participants included in any comparisons receiving similar treatment/care, other than the exposure or intervention of interest?; Q4 = Was there a control group?; Q5 = Were there multiple measurements of the outcome both pre and post the intervention/exposure?; Q6 = Was follow up complete and, if not, were differences between groups in terms of their follow up adequately described and analyzed?; Q7 = Were the outcomes of participants included in any comparisons measured in the same way?; Q8 = Were outcomes measured in a reliable way?; Q9 = Was appropriate statistical analysis used? The answer to each question was: yes, no, or not applicable. Domain-level reports provide the basis for an overall risk-of-bias judgment; the presence of one risk of bias was interpreted as an overall serious risk of bias for that study.

## 3. Results

**Figure 1:**
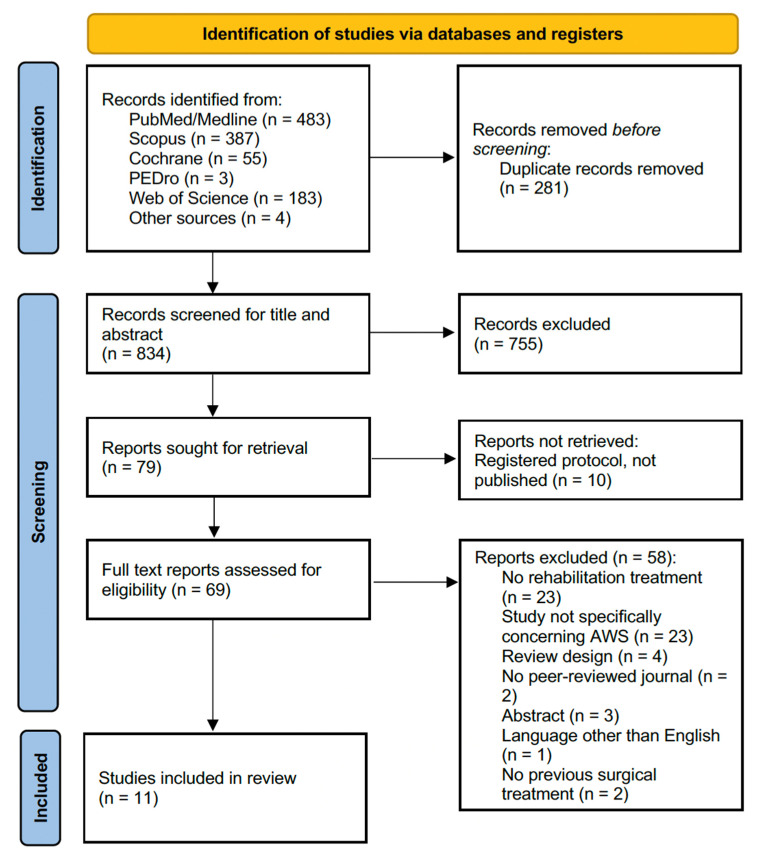
PRISMA 2020 flow chart.

The search strategy identified 1115 records from the five databases and 4 records from other sources (reference lists of the included studies). Figure 1 shows the PRISMA 2020 flow diagram of the search process.

**Figure 2:**
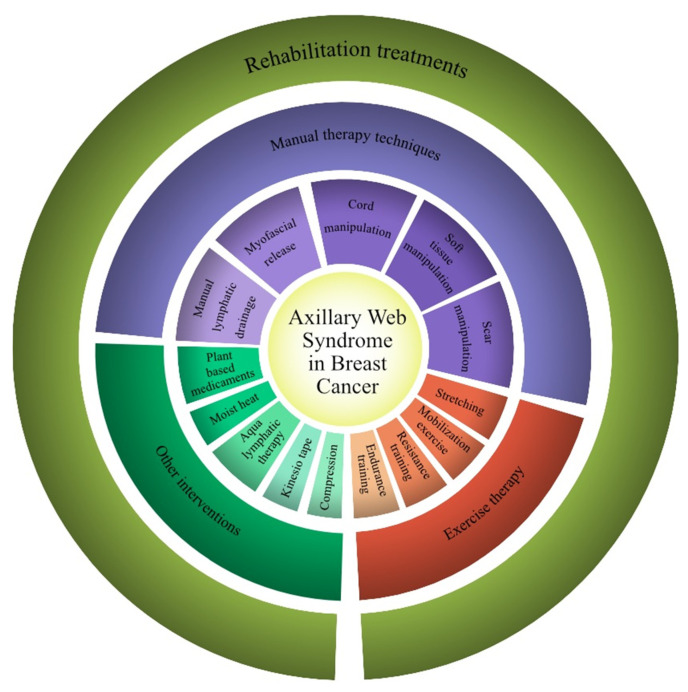
Rehabilitation therapy interventions for axillary web syndrome proposed in this systematic review.

After duplicate removal, 834 studies were assessed for eligibility and screened for title and abstract. After excluding 755 records, 79 full-text records were assessed for eligibility. On the other hand, ten reports were not retrieved because they were registered protocols not published yet. Fifty-eight articles were excluded for inconsistency with the eligibility criteria (twenty-three studies did not propose a rehabilitation treatment, twenty-three studies did not explicitly concern AWS, four had a review design, two were not published in peer-reviewed journals, three were abstracts, one was written in a language other than English, and in two studied BC patients who did not undergo surgery). The studies assessed in full text and the reasons for exclusions are presented in detail in Figure 2.

As a result, 11 studies were included in the present work [13,31,32,33,34,35,36,37,38,39,40]. 

The studies included were two (18.2%) RCTs [31,32], one (9.1%) non-RCT [33], one (9.1%) retrospective observational study [34], and seven (63.6%) case reports [13,35,36,37,38,39,40].

The publication year of the studies included ranged between 2006 [34] and 2020 [13], while the countries of origin of the studies included in this systematic review were as follows: three studies (27.3%) were conducted in the USA [34,35,39], one (9.1%) was conducted in Canada [38], two studies (18.2%) were conducted in Europe (one conducted in Belgium [33] and one in Italy [13]), three studies (27.3%) were conducted in Asia (one conducted in South Korea [31], one in China [37], and one in Israel [40]), and one (9.1%) was conducted in Egypt [32]. The remaining (9.1%) study was an international collaboration [36]. 

In the present review, 174 subjects were assessed in the included studies, all females (100%), with overall dropouts of 13 patients. The ages of the subjects included ranged from 37 [38] to 66 [13]. Of note, Moreau et al. [33] and Wyrick et al. [34] did not report the age of the study participants. BMI was reported in three studies: the case reports of de Sire et al. [13] and Jacob et al. [40] (BMI of 22 kg/m^2^ and 23.4 kg/m^2^, respectively), and the RCT by Cho et al. [31] (n = 15 with a BMI ≥ 25 kg/m^2^; n = 26 with a BMI < 25 kg/m^2^).

The time between breast surgery procedures and rehabilitation intervention ranged between 3 days [37] and 1 year [33,39]. The surgical treatments received were mastectomy (n = 42), lumpectomy (n = 21), double lumpectomy (n = 1), quadrantectomy (n = 1), breast reconstruction surgery (n = 13), axillary lymph node dissection (n = 86), and sentinel node biopsy (n = 9), while chemotherapy, radiotherapy, and hormonal therapy were performed on 33, 54, and 12 patients, respectively [13,31,32,33,34,35,36,37,38,39,40]. Three studies [31,32,33] assessed rehabilitation treatment compared with other treatments. 

The sample characterization of each study included is summarized in Table 2.

### 3.1. Quality Assessment

Table 3 reports the quality assessment of the study included for each domain of the JBI-QES (non-randomized experimental studies) [30].

All studies had at least one serious risk of bias, with a consequent overall serious risk of bias for those studies. Most of the included studies (n = 7) were case reports [13,35,36,37,38,39,40] with the intrinsic limitations of lack of control and of statistical analysis. Measurements were not performed more than once pre- and post-intervention in five studies [31,32,34,37,39], and only four studies [13,33,35,39] reported follow-up data. Lastly, statistical analysis was not appropriate due to the lack of intention-to-treat analysis in the two RCTs [31,32] and the non-RCT [33] included.

### 3.2. Rehabilitation Therapy Interventions 

The rehabilitative treatments proposed for AWS varied among the studies included and were proposed in different combinations. In more detail, different manual therapy techniques were assessed [13,31,32,33,34,35,36,37,38,39,40], including MLD [13,31,33,40] (in more detail, the Vodder method [31] and Leduc method [33]), myofascial release techniques [13,32,35], cord manipulation [13,31,33,35,37,38,40], soft tissue manipulation [13,31,32,33,34,35,36,39], and scar manipulation [13,35,37,40]. 

On the other hand, several rehabilitation programs included exercise therapies [13,31,33,34,35,36,37,38,39,40]. In particular, eight studies assessed stretching exercises [13,31,34,35,36,38,39,40], four studies assessed resistance training [13,31,34,37], six studies assessed mobilization exercises [31,33,34,36,38,39], and one study assessed a combination of endurance and resistance training [40]. 

Lastly, other rehabilitative interventions included Kinesio tape [32], compression bandages and intermittent pneumatic compression [34], compression garments [40], aqua lymphatic therapy [40], and moist heat applied to the axilla and inner arm [38].

Interestingly, Wei et al. [37] combined rehabilitation treatment with the oral administration of a plant-based medicament (Aesculus hippocastanum, 300 mg) twice a day. 

All the rehabilitation treatments identified by this systematic review are shown in Figure 2.

A trained physical therapist supervised the rehabilitative treatments in nine studies (90%) [13,31,32,33,34,35,36,38,39]. In contrast, three studies (30%) [13,31,32] offered supervised therapy only, while six studies (60%) [33,34,35,36,38,39] combined supervised rehabilitation with home-based treatment. Lastly, one study (10%) [37] proposed only a home-based rehabilitation.

The number of sessions per week ranged between 2 [32,36,38] and 4 [39], while the mean rehabilitation program duration ranged between 3 weeks [13,38] and 10.1 ± 9.5 weeks [34]; when specified, the duration of each intervention lasted from 30 min [31,36,37] to 60 min [40].

Follow-up was described in four studies. In particular, one study performed a follow-up at three months [39], one study at two months and one year [35], one study at one year [13], and one study until resolution [33]. 

### 3.3. Main Findings 

Self-reported pain was assessed in seven studies [13,31,32,33,37,39,40], and all of them reported improvement in pain intensity [13,31,32,33,37,39,40]. In more detail, four studies [32,33,37,40] assessed self-reported pain intensity with a visual analogue scale (VAS). The RCT by Ibrahim et al. [32] reported a significant (*p* = 0.0001) improvement in the VAS in all groups, with no intergroup differences (*p* = 0.31) [32]. Similarly, the non-RCT by Moreau et al. [33] showed a significant improvement in VAS scores in both groups (*p* < 0.05) without reporting significant differences between groups (*p* = NR) [33]. Lastly, in the case reports by Wei et al. [37] and Jacob et al. [40], the VAS decreased, respectively, from 7 to 0 [37] and from 8 to 0 [40]. 

On the other hand, three studies [13,31,39] assessed pain intensity with a numerical pain rating scale (NPRS/NRS). In particular, the RCT by Cho et al. [31] underlined a significant improvement in NRS scores in both intervention groups after the 4-week intervention (*p* < 0.05). Interestingly, their intergroup NRS scores were significantly lower in the group treated with physical therapy combined with MLD, compared to the group that performed physical therapy only (*p* < 0.05) [31]. Lastly, both de Sire et al. [13] and Crane et al. [39] presented a case report with NPRS improvement from 5 to 0 in 3 weeks and from 5 to 1 in 4 weeks, respectively. 

Upper limb function was assessed in ten studies. In particular, nine studies [13,31,33,34,35,36,37,38,39] assessed shoulder joint range of motion, reporting positive results after the intervention [13,31,33,34,35,36,37,38,39]. The RCT by Cho et al. [31] and the non-RCT by Moreau et al. [33] reported a significant (*p* < 0.05) improvement in ROM in all groups without highlighting intergroup differences (*p* = NR) [31,33]. Similarly, Wyrick et al. [34] reported an improvement in shoulder ROM (mean improvements: 52 ± 21° in abduction; 39 ± 20° in flexion) after four weeks of rehabilitation treatment. All the case reports underlined ROM shoulder improvements [13,34,35,36,37,38,39]. Differently, the Disability of the Arm, Shoulder and Hand (DASH) score and the Short Form (QuickDASH) score were assessed in three studies [13,31,35]. In more detail, Cho et al. [31] reported a significant (*p* < 0.05) improvement in DASH scores in all groups without showing intergroup differences (*p* = NR) [31]. Similarly, Lattanzi et al. [35] reported a DASH score improvement from 32.5 to 7.5, while the case report by de Sire et al. [13] underlined a QuickDASH score improvement from 40 to 0. On the other hand, the Patient-Specific Functional Scale (PSFS) was assessed in one study [39], reporting positive results.

Lastly, muscle strength was assessed with a hand-held dynamometer by Cho et al. [31], underlining significant improvements after the intervention (*p* < 0.05). 

See Table 2 for further details on the rehabilitative approaches and main findings of the included studies.

HR-QoL represents a secondary outcome of the present work and was assessed in two studies [13,31]. In more detail, the RCTs by Cho et al. [31] assessed HR-QoL with the Breast Cancer-Specific Quality of Life Questionnaire and the European Organization for Research and Treatment of Cancer Core, reporting a significant (*p* < 0.05) improvement in function and symptom scores in all groups, with no differences between groups (*p* = NR) [31]. Similarly, de Sire et al. [13] assessed HR-QoL with the European Quality of Life, 5 Dimensions, 3 Levels (EQ-5D-3L) questionnaire, and the European Quality of Life Visual Analogue Scale (EQ-VAS). The authors showed an increase in both indexes (EQ-5D-3L: 0.662 vs. 1.000; EQ-VAS: 75 vs. 90) [13].

The resolution of the clinical presentation was studied in nine studies [31,32,33,34,35,36,37,38,40]; however, a wide variety of methods were used to assess clinical resolution, in particular cord evaluation [31,32,34,35,37,38,40], adherence evaluation [33], and tissue movement and glide measurements [36]. Interestingly, Cho et al. [31] reported that arm volumes were significantly lower in the group treated with manual lymphatic drainage and physical therapy compared to the physical therapy-only group (*p* < 0.05). Furthermore, no lymphedema was observed in the first group in contrast with the second group, underlining significant differences between groups (*p* < 0.05). On the other hand, the percentage of visible cords was not significantly different between the two groups (28.5% vs. 35%, *p* = 0.658) [31]. Interestingly, Ibrahim et al. [32] found a significant decrease in the thickness of the cords in each group after the treatment (*p* < 0.05); however, there were no significant differences between the three groups (*p* = 0.39). In contrast, cord disorganization significantly improved after the rehabilitation treatment in each group (*p* < 0.05). Moreover, there was a significant difference between the group receiving direct myofascial release combined with Kinesio tape treatment compared to the direct myofascial release-only group (*p* = 0.03) and the Kinesio tape-only group (*p* = 0.009). No significant differences were reported between the myofascial release-only group and the Kinesio tape-only group (*p* = 0.08) [32].

Intriguingly, Wyrick et al. [34] assessed differences between the irregular attendance patients (who missed more than two consecutive weeks of therapy) and regular attendance patients. The authors reported significant differences between groups in terms of treatment duration (18.0 ± 17.1 vs. 7.3 ± 3.4 weeks; *p* = 0.012) [34]. 

## 4. Discussion

The recent increase in the overall survival of BC women has pointed out the need to address the growing issue of sequelae in BC survivors, as well as their assessment and treatment [3]. In this scenario, AWS is one of the most common post-surgical sequelae affecting both the physical function and HR-QoL of BC survivors [41]. Despite the high incidence of the disease [19,20], the optimal therapeutic management of AWS is still debated, and strong evidence supporting rehabilitation treatment for pain relief and functional improvement in AWS patients is lacking [26,42]. Therefore, this paper summarized the state of art of the current rehabilitation intervention proposed in AWS management to provide a broad overview of the potential treatment for this burdensome condition. 

The main findings of the present systematic review underline the high heterogeneity of rehabilitation interventions that might positively influence AWS symptoms, improving the cord characteristics and the functional limitations affecting the quality of life of BC female patients [13,31,32,33,34,35,36,37,38,39,40]. These data are in accordance with the current trend in rehabilitation management of other BC sequelae, highlighting the need for integrated therapeutic strategies improving functional outcomes and HR-QoL with a multitarget intervention [43,44].

Our review identified two randomized controlled trials assessing the effects of a different combination of rehabilitation modalities in AWS patients [31,32]. In these clinical trials, manual therapy (myofascial release, manual lymphatic drainage, soft tissue mobilization), resistance exercises, mobilization, stretching, and Kinesio taping were performed in an integrated rehabilitation program with positive results [31,32]. 

In more detail, Cho et al. [31] reported significant differences between physical therapy and physical therapy combined with MLD. Moreover, the authors underlined an upper limb volume reduction in the treatment arm after MLD, as reported by previous studies assessing BCRL patients [45,46]. In accordance, the non-RCT by Moreau et al. [33] reported a significant improvement after a rehabilitation protocol including MLD and light stretching, despite no significant differences being reported in the between-group analysis. These controversial results emphasize the notion that more studies are needed to confirm the role of MLD in patients with AWS. On the other hand, MLD might be considered in a multitarget rehabilitation strategy covering different pathological targets in BC survivors, despite several questions still being open about its effectiveness in both the management and prevention of BCRL [47,48,49]. 

On the other hand, the RCT by Ibrahim et al. [32] assessed the role of direct myofascial release, Kinesio tape, and the combination of both, without reporting differences in terms of AWS clinical resolution. Besides the significant intragroup differences reported in each treatment arm, no intergroup differences were shown in terms of pain relief between patients undergoing different rehabilitation treatments. Therefore, it is particularly difficult to draw any strong conclusion about the impact of different rehabilitation treatments to treat AWS, and specific rehabilitation programs supported by strong evidence are lacking. 

Interestingly, positive effects of manual therapy and exercise therapy were reported by most of the studies included in the present review [13,33,34,35,36,37,38,39,40], highlighting promising effects of a comprehensive rehabilitation treatment, although comparative data about the different rehabilitation strategies are lacking. Moreover, the low quality of these studies severely affects the clinical implications of our results. 

In this context, the recent systematic review by Luz et al. [42] emphasized the need for high-quality studies focusing on the low number of studies assessing conservative treatments to manage this neglected issue. In particular, the authors included just four studies with heterogeneous designs, providing low-quality evidence supporting the optimal therapeutic intervention in the complex management of AWS. In contrast, Agostini et al. [26] recently reviewed the therapeutic approach to AWS in the rehabilitation setting. In this narrative review, the authors underlined the positive role of soft tissue techniques, therapeutic exercises, and muscle strengthening in reducing the disabling consequences of AWS. However, the authors stated that the lack of a systematic approach might limit the strength of the conclusions. Lastly, Yeung et al. [14] in 2015 systematically reviewed the literature deeply characterizing AWS clinical presentation. However, the authors assessed the epidemiology, etiology, risk factors, and consequences of this disabling disease, without focusing on rehabilitation treatments and without providing evidence for optimal AWS management.

In this scenario, a growing amount of literature is now focusing on individualized therapeutic approaches to address cancer’s long-term consequences of survivorship issues [9,50]. In more detail, HR-QoL is currently considered an important part of the healthcare system and a cornerstone of modern treatment strategies [51,52]. It has been proposed that a precision medicine approach might be mandatory not only for breast cancer diagnosis and treatments but also for the long-term consequences affecting breast cancer quality of life [7,50,53,54]. Therefore, the urgent need for a tailored treatment based on patients’ characteristics reflects the current innovation in the translational field based on cutting-edge technologies improving the complex management of breast cancer survivors, including distinct approaches and different specialists [51,53,54,55,56].

On the other hand, besides these advances, AWS is still far from being fully characterized, and several questions about its etiology are still open [13,14]. In this context, the precise approach to AWS might be improved by a deeper understanding of the mechanisms underpinning this condition and should be based on a translational approach involving a multidisciplinary team to characterize the disease better. In this scenario, the incidence of AWS is higher in patients who underwent excision of a greater number of lymph nodes [23], which is also a widely known risk factor for the development of BCRL. Thus, the interruption of axillary lymphatic ducts appears to play a crucial role in AWS development. This hypothesis was supported in the study by Moskovitz et al. [12], who found a few cases of AWS after breast surgery in the absence of axillary node dissection. 

Altogether, the results of the present work underline that the optimal therapeutic characteristics in terms of treatment modality, intensity, session duration, and treatment duration are far from being fully understood. Moreover, our findings show that the large gap in knowledge about the optimal treatments might be partly related to the small number of studies assessing this underestimated condition and partly related to the low level of evidence available. We reported evidence that comes largely from case report studies that represent 63.6% of the studies included in the present work [13,35,36,37,38,39,40]. Moreover, several limitations have been identified in the studies included. In more detail, the non-RCT by Moreau et al. [33] did not report any methods of allocation of the study participants, in addition to failing to report information about the size of each intervention group; furthermore, sample characteristics were not provided in detail [33]. On the other hand, blinding of the rehabilitation treatment represents an intrinsic limitation of several studies included [31,32,33]; only Cho et al. [31] reported the blinding of the operator who performed the rehabilitation treatment [31]. Furthermore, no author performed an intention-to-treat analysis or reported any possible dropouts in the reported data [31,32,33]. 

Besides the intrinsic limitations of the studies included in the present work, we are aware that our systematic review is not free from limitations. In particular, the low number of RCTs included severely affects the strength of our findings. In addition, most of the studies included assessed the role of combined therapy; therefore, it is difficult to draw precise indications about the role of each individual treatment modality in AWS symptom management. 

However, to the best of our knowledge, the present paper represents the first systematic review reporting the state of the art of rehabilitation in AWS, focusing on differences between different approaches.

## 5. Conclusions

At the present time, several rehabilitative treatments have been proposed to reduce pain and improve functional outcomes in AWS patients. To date, the optimal rehabilitation treatment is far from being fully characterized, albeit different protocols might be considered as safe therapeutic interventions included in a wider rehabilitation approach aimed at improving functioning in patients with AWS. 

On the other hand, the present systematic review also highlights the need for good-quality studies to investigate the effects of specific rehabilitative interventions in AWS patients.

A translational approach characterizing the pathophysiological mechanisms underpinning AWS is mandatory to improve the definition of a patient-tailored rehabilitation plan for this burdensome condition.

## Figures and Tables

**Table 1:** Search strategy.

***PubMed:*** ((axillary web syndrome) OR (AWS) OR (cords) OR (cording) OR (webbing) OR (lymphatic cord) OR (axillary web cord) OR (vascular string) OR (lymphatic thrombosis) OR (lymphatic cording) OR (lymphatic cord) OR (axillary string) OR (axillary web) OR (vascular string) OR (mondor disease) OR (axillary web cord) OR (axilla band) OR (axilla cord) OR (fibrous banding) OR (fibrotic bands) OR (string phenomenon) OR (cording lymphoedema) OR (superficial lymphatic thrombosis) OR (vascular ring) OR (fibrous cords) OR (lymph vessel fibrosis) OR (syndrome of the axillary cords)) AND ((breast cancer) OR (breast tumor) OR (breast malignancy) OR (breast neoplasm)) AND ((treatment) OR (therapy) OR (physiotherapy) OR (rehabilitation) OR (exercise) OR (exercises) OR (therapeutic exercise) OR (physical exercise) OR (management))
***Scopus:*** TITLE-ABS-KEY ((((AWS) OR (axillary AND web AND syndrome) OR (lymphatic AND cord) OR (axillary AND web AND cord) OR (vascular AND string) OR (lymphatic AND coding) OR (axillary AND string) OR (axillary AND web) OR (vascular AND string) OR (axilla AND band) OR (axilla AND cord) OR (fibrous AND banding) OR (fibrotic AND band) OR (string AND phenomenon) OR (coding AND lymphoedema) OR (vascular AND ring) OR (fibrous AND cords) OR (lymph AND vessel AND fibrosis) OR (syndrome AND of AND the AND axillary AND cords)) AND ((breast AND cancer) OR (breast AND tumor) OR (breast AND malignancy) OR (breast AND neoplasm)) AND (treatment OR exercise OR (physical AND exercise) OR physiotherapy OR rehabilitation OR management OR (therapeutic AND exercise))))
***Cochrane Central Register of Controlled Trials (CENTRAL):*** ((axillary web syndrome) OR (AWS) OR (cords) OR (cording) OR (webbing) OR (lymphatic cord) OR (axillary web cord) OR (vascular string) OR (lymphatic thrombosis) OR (lymphatic cording) OR (lymphatic cord) OR (axillary string) OR (axillary web) OR (vascular string) OR (mondor disease) OR (axillary web cord) OR (axilla band) OR (axilla cord) OR (fibrous banding) OR (fibrotic bands) OR (string phenomenon) OR (cording lymphoedema) OR (superficial lymphatic thrombosis) OR (vascular ring) OR (fibrous cords) OR (lymph vessel fibrosis) OR (syndrome of the axillary cords)):ti,ab,kw AND ((breast cancer) OR (breast tumor) OR (breast malignancy) OR (breast neoplasm)):ti,ab,kw AND ((treatment) OR (therapy) OR (physiotherapy) OR (rehabilitation) OR (exercise) OR (exercises) OR (therapeutic exercise) OR (physical exercise) OR (management)):ti,ab,kw
***Physiotherapy Evidence Database (PEDro):*** axillary web syndrome*treatment
***Web of Science:*** TS = (AWS OR (axillary web syndrome) OR (lymphatic cord) OR (axillary web cord) OR (vascular string) OR (lymphatic coding) OR (axillary string) OR (axillary web) OR (vascular string) OR (axilla band) OR (axilla cord) OR (fibrous banding) OR (fibrotic band) OR (string phenomenon) OR (coding lymphoedema) OR (fibrous cords) OR (lymph vessel fibrosis) ) AND (breast cancer OR breast tumor) AND (treatment OR exercise OR (physical exercise) OR physiotherapy OR rehabilitation OR management OR (therapeutic exercise))

**Table 2:** Main characteristics of the studies included.

Author Year Country	Design	Participants		Time from Surgery	Breast Cancer Treatment	Intervention Modality	Frequency, Volume, Intensity, Protocol Duration	Control	Outcomes	Main Findings
		*Sample Size (Dropouts)*	*Mean age* (years) *BMI* (kg/m^2^*)*							
Cho et al., 2015 [31] South Korea	RCT	48 (7) PTMLD: n = 21 PT: n = 20	PTMLD: 46.6 ± 6.8 PT: 50.7 ± 9.6 PTMLD: ≥25: n = 6; <25: n = 15 PT: ≥25: n = 9; <25: n = 11	At least 4 weeks	PTMLD: mastectomy (n = 12), lumpectomy (n = 7), breast reconstruction (n = 2). Chemotherapy (n = 9), radiotherapy (n = 21), hormone therapy (n = 14). PT: mastectomy (n = 16), lumpectomy (n = 3), breast reconstruction (n = 1). Chemotherapy (n = 11), radiotherapy (n = 19), hormone therapy (n = 12).	PTMLD: PT consisting of 10 min warm-up (stretching); shoulder flexor, shoulder abductor, and elbow flexor strengthening exercises; 30 min of manual therapy: soft tissue mobilization techniques and stretching for tight tissue cords, shoulder abduction, elbow extension, and wrist supination and extension stretching exercises, shoulder girdle mobilization; and PROM exercises; 10 min cooldown (stretching) + MLD Vodder method 30 min for session. Supervised.	PTMLD: PT 3 times/week (50 min per session) for 4 weeks + MLD 5 times/week (30 min per session) for 4 weeks. PT: PT 3 times/week (50 min per session) for 4 weeks. PT intensity: 60–80% 1RM. MLD intensity: from comfortable to mild discomfort.	PT consisting of 10 min warm-up (stretching); shoulder flexor, shoulder abductor, and elbow flexor strengthening exercises; 30 min of manual therapy: soft tissue mobilization techniques and stretching for tight tissue cords, shoulder abduction, elbow extension, and wrist supination and extension stretching exercises, shoulder girdle mobilization; and PROM exercises; 10 min cooldown (stretching).	Arm volume: circumference measurements; muscular strength: hand-held dynamometer; AROM: digital inclinometer; DASH; EORTC QLQ-C30; EORTC QLQ-BR23; NRS.	This study reports about 48 post-surgical BC AWS patients (mean age PTMLD: 46.6 ± 6.8, PT: 50.7 ± 9.6). They were treated 4 weeks after surgery. The main finding is represented by the improvement observed in QOL (both EORTC QLQ-C30; EORTC QLQ-BR23) including functional and symptom aspects, shoulder flexor strength, and AROM, DASH, and NRS scores that were significantly improved in both groups after the 4-week intervention (*p* < 0.05). NRS score and arm volume were significantly lower in the PTMLD group than in the PT group (*p* < 0.05). No lymphedema was observed in the PTMLD group but was observed in the PT group (*p* < 0.05). Visible cords percentage was not significantly different between the two groups (28.5% PTMLD, 35% PT, *p* = 0.658).
Ibrahim et al., 2018 [32] Egypt	RCT	60 (NA) Group A: n = 20 Group B: n = 20 Group C: n = 20	40–50 NA	3 months	ALND	Group A: direct myofascial release (in shoulder abduction) and Kinesio tape (in shoulder abduction). Supervised.	All groups: 2 sessions/week for 4 weeks Volume: NR Intensity: NR	Group B: direct myofascial release (in shoulder abduction). Group C: Kinesio tape (in shoulder abduction).	VAS, ultrasound for assessment of AWS cord thickness and disorganization.	This study reports about 60 post-surgical BC AWS patients (aged between 40 and 50 years). They were treated 3 months after surgery. The main finding is represented by the improvement observed in VAS (each group had a significant decrease post-treatment, *p* = 0.0001, but there was no significant difference between the three groups, *p* = 0.31); in decrease thickness of the cords (each group had a significant decrease post-treatment, *p* < 0.05, but there was no significant difference between the three groups, *p* = 0.39); and in cord disorganization (each group had a significant improvement post-treatment, *p* < 0.05, and there was a significant difference between groups A and B, *p* = 0.03, and A and C, *p* = 0.009, while there was no significant difference between B and C, *p* = 0.08).
Moreau et al., 2010 [33] Belgium	Non-RCT	28 (NA) Group 1: n = NA Group 2: n = NA	NA NA	NA	BC surgery with axillary clearing in 82.14% of cases (n = 23), and only sentinel node removal in 17.85% of cases (n = 5).	Group 1: MLD (Leduc method) + light adherence stretch (according to ROM, with no pain). Group 2: soft tissue work + adherence stretch (petrissage method, pain could be elicited) + upper extremity mobilization. Supervised.	All groups: at least 13 sessions. Frequency: NR Volume: NR Intensity: NR	NR	Adherence evaluation, upper extremities ROM, VAS.	This study reports about 28 post-surgical BC AWS patients (mean age not specified). The main finding is represented by the improvement observed in adherences, upper extremities ROM, and VAS, which resulted in being significant after 13 sessions of treatment (*p* < 0.05), while no significant difference was present between groups.
Wyrick et al., 2006 [34] United States	Retrospective observational study	31 (6)	NA NA	From 14 days to 5.8 years; 17% of patients > 1 year.	Lumpectomy (n = 7), double lumpectomy (n = 1), lumpectomy followed by mastectomy (n = 4), mastectomy (n = 8), mastectomy followed by later breast reconstruction (n = 4), mastectomy followed by immediate breast reconstruction (n = 6). One patient was seen twice but had only one surgical procedure. Concomitant cancer therapy (chemotherapy or radiation, n = 12).	Home exercises program with active mobilization; therapeutic exercise, including soft tissue stretching, progressive resistance exercises, Airdyne Bicycle training; if patients had lymphedema or persistent swelling, manual therapy, compression bandages, and intermitting pneumatic compression were also used. Supervision not specified.	Average duration of treatment: 10.1 ± 9.5 weeks. Frequency: NR Volume: NR Intensity: NR	NR	Cording severity (mild, mild to moderate, moderate, moderate to severe, severe), ROM, length of care.	This study reports about 31 post-surgical BC AWS patients (mean age not specified). They were treated from 14 days to 5.8 years after surgery. The main finding is represented by the resolution in lymphatic cording, which was faster with physical therapy. Shoulder ROM improved after 4 weeks of treatment (abduction improved by 52 ± 21° and flexion improved by 39 ± 20°). The difference in treatment duration between regular and irregular patients was statistically significant (*p* = 0.012): duration of treatment for regular patients (n = 18) was 7.3 ± 3.4 weeks (less than 2 months); duration of treatment for irregular patients (n = 7) was 18.0 ± 17.1 weeks; mean treatment duration for patients with concomitant cancer therapy leading to cancellations (n = 12) was 17.0 ± 14.8 weeks.
Lattanzi et al., 2012 [35] United States	Case report	1	44 NA	10 days.	Lumpectomy and sentinel node dissection. Thirty-five sessions of radiotherapy.	First week: soft tissue mobilization of cords, skin traction techniques, and myofascial release techniques. Second week: + two-person cord traction technique, self-elongation techniques, and skin traction and scar massage. Home stretching program. Supervised.	Physical/occupational therapy 3 times/week for 2 weeks followed by pause during 35 radiotherapy treatments. Then, 2 times/week for 1 week, and then 1 time/week for 2 weeks (total of 5 weeks of protocol duration). Volume: NR Intensity: NR.	NR	ROM, cords evaluation, muscle strength and function, DASH.	This case report presents 1 post-surgical BC AWS patient (44 years old). She was treated 10 days after surgery. The main finding is represented by the improvement in ROM, cords, muscle strength and function, and DASH score (32.5 vs. 7.5).
Fourie et al., 2009 [36] South Africa, United Kingdom	Case report	1	47 NA	22 days.	Left modified radical mastectomy with removal of six axillary lymph nodes.	Manual soft tissue techniques. Home program of gentle stretching and self-mobilization. Supervised.	First week: 2 sessions; Days 8–10: 1 session a day; Up to 26 days: 6 sessions. Total of 11 sessions. 30–45 min each session. Intensity: NR.	NR	AROM, PROM, tissue movement and glide.	This case report presents 1 post-surgical BC AWS patient (47 years old). She was treated 22 days after surgery. The main finding is represented by the improvement in AROM, PROM, tissue movement and glide, until full range of movement with no visible or palpable cording.
Wei et al., 2013 [37] China	Case report	1	39 NA	3 days.	Breast-conserving surgery and axillary lymph node biopsy; 17 days later secondary breast-conserving surgery.	Home program: shoulder exercises and massage to the cord-like structure. Non-supervised.	Plant-based medicament (Aesculus hippocastanum) 300 mg twice a day. Physical program every day in the morning and at bedtime for 30 min, for 3 weeks. Intensity: NR	NR	ROM, VAS, cords evaluation both manual and ultrasound.	This case report presents 1 post-surgical BC AWS patient (39 years old). She was treated 3 days after surgery. The main finding is represented by the improvement after 3 weeks in ROM (90° vs. 170°), VAS (7 vs. 0), cords that became invisible and non-palpable (both manually and by ultrasound). Numbness and tightness were still present but diminished. The plant-based medication Aesculus hippocastanum could have played a role.
Tilley et al., 2009 [38] Canada	Case report	1	37 NA	1 week.	Lumpectomy, sentinel lymph node biopsy, and ALND for a node-positive BC.	Moist heat to the axilla and inner arm for 10 min per session. Shoulder flexion and abduction ROM exercises and gentle stretching. Cord stretching. Home exercises (gentle arm flexion and horizontal abduction). Supervised.	6 physiotherapy sessions in 3 weeks period. Volume: NR Intensity: NR	NR	ROM, cords evaluation.	This case report presents 1 post-surgical BC AWS patient (37 years old). She was assessed 1 week after surgery. The main finding is represented by the improvement in ROM (135° and 123° vs. 180° and 180°, flexion and abduction, respectively). The cord improved but was still palpable at the end of her treatment sessions, 7 weeks after surgery.
de Sire et al., 2020 [13] Italy	Case report	1	66 22	1 month.	Left inner upper quadrantectomy with negative sentinel node biopsy.	Manual therapy: myofascial release techniques with soft-tissue mobilization, massage and manipulation of the tight cord and scar tissues, therapeutic shoulder exercises including stretching, and MLD. Supervised.	Fondaparinux 2.5 mg/day for 3 weeks. Rehabilitation program 3 times/week for 3 weeks, 45 min per session. Intensity: NR	NR	ROM, NPRS, Quick DASH, EQ-5D-3L index, EQ-VAS.	This case report presents 1 post-surgical BC AWS + Mondor’s disease patient (66 years old). She was treated 1 month after surgery. The main finding is represented by the improvement after 3 weeks treatment in shoulder ROM (80° and 100° vs. 170° and 170°, flexion and abduction, respectively), NPRS (5 vs. 0), Quick DASH (40 vs. 0), EQ-5D-3L index (0.662 vs. 1.000), EQ-VAS (75 vs. 90).
Crane et al., 2017 [39] United States	Case report	1	48 NA	1 year.	Bilateral mastectomy and negative lymph node dissection. Following surgery, a course of chemotherapy. Three months after completion of chemotherapy, bilateral latissimus dorsi flap reconstruction.	IASTM to the axilla at end range abduction for 5 min, thoracic/thoracolumbar junction manipulation, and flexibility exercise. Home exercise program consisting of shoulder girdle and thoracic stretching. Supervised.	4 times/week for 4 weeks. Home exercises 3 times/day each day. Volume: NR Intensity: NR	NR	ROM, NPRS, PSFS, thoracic rotation.	This case report presents 1 post-surgical BC AWS patient (48 years old). She was treated 1 year after surgery. The main finding is represented by the improvement in shoulder ROM (140° and 150° vs. 178° and 174°, flexion and abduction, respectively), bilateral thoracic rotation (25% vs. 100%), NPRS (5 vs. 1), and PSFS (19/30 vs. 30/30).
Jacob et al., 2019 [40] Israel	Case report	1	65 23.4	7 months	Left breast lumpectomy + ALND + intraoperative radiation therapy and whole breast radiation.	MLD, scar tissue techniques, cord stretching, self-massage, supportive bras. Home program: self-lymph-massage, compression garments, stretching exercise, Tidhar method of aqua lymphatic therapy, physical activity program. Supervised.	1 time/week for 6 weeks, 60 min per session. Intensity: NR	NR	ROM, VAS during shoulder ROM, cording evaluation.	This case report presents 1 post-surgical BC AWS patient (65 years old). She was treated 7 months after surgery. The main finding is represented by the improvement in VAS during shoulder ROM (8 vs. 0), which was never limited, and the disappearance of cording at the end of treatment.

Abbreviations: 1RM: one repetition maximum; ALND: axillary lymph node dissection; AROM: active range of motion; AWS: axillary web syndrome; BC: breast cancer; BMI: body mass index; DASH: Disability of the Arm, Shoulder and Hand; EORTC QLQ-BR23: Breast Cancer-Specific Quality of Life Questionnaire; EORTC QLQ-C30: European Organization for Research and Treatment of Cancer Core; EQ-5D-3L: European Quality of Life, 5 Dimensions, 3 Levels; EQ-VAS: European Quality of Life Visual Analogue Scale; IASTM: instrument-assisted soft tissue mobilization; MLD: manual lymphatic drainage; NA: not applicable; NPRS/NRS: numerical pain rating scale; NR: not reported; PROM: passive range of motion; PSFS: patient-specific functional scale; PT: physical therapy; PTMLD: physical therapy combined with manual lymphatic drainage; QOL: quality of life; QuickDASH: Disabilities of the Arm, Shoulder and Hand Questionnaire, Short Form; RCT: randomized controlled trial; ROM: range of motion; VAS: visual analogue scale.

**Table 3:** Joanna Briggs Institute Critical Appraisal Checklist for Quasi-Experimental Studies (non-randomized experimental studies).

Author and Year	Q1	Q2	Q3	Q4	Q5	Q6	Q7	Q8	Q9
de Sire et al., 2020 [13]	Y	N/A	N/A	N/A	Y	Y	N/A	Y	N/A
Cho et al., 2015 [31]	Y	Y	Y	Y	N	N	Y	Y	N
Ibrahim et al., 2018 [32]	Y	Y	Y	Y	N	N	Y	Y	N
Moreau et al., 2010 [33]	Y	Y	Y	Y	Y	Y	Y	Y	N
Wyrick et al., 2006 [34]	Y	N/A	N/A	N	N	N	Y	Y	Y
Lattanzi et al., 2012 [35]	Y	N/A	N/A	N/A	Y	Y	N/A	Y	N/A
Fourie et al., 2009 [36]	Y	N/A	N/A	N/A	Y	N	N/A	Y	N/A
Wei et al., 2013 [37]	Y	N/A	N/A	N/A	N	N	N/A	Y	N/A
Tilley et al., 2009 [38]	Y	N/A	N/A	N/A	Y	N	N/A	Y	N/A
Crane et al., 2017 [39]	Y	N/A	N/A	N/A	N	Y	N/A	Y	N/A
Jacob et al., 2019 [40]	Y	N/A	N/A	N/A	Y	N	N/A	Y	N/A

Question Q1 = Is it clear in the study what is the ‘cause’ and what is the ‘effect’ (i.e., there is no confusion about which variable comes first)?; Q2 = Were the participants included in any comparisons similar?; Q3 = Were the participants included in any comparisons receiving similar treatment/care, other than the exposure or intervention of interest?; Q4 = Was there a control group?; Q5 = Were there multiple measurements of the outcome both pre and post the intervention/exposure?; Q6 = Was follow up complete and, if not, were differences between groups in terms of their follow up adequately described and analyzed?; Q7 = Were the outcomes of participants included in any comparisons measured in the same way?; Q8 = Were outcomes measured in a reliable way?; Q9 = Was appropriate statistical analysis used?; N = no; Y = yes; N/A = not applicable.

## Data Availability

The datasets generated during the current study are available from the corresponding author on reasonable request.
